# Cardiac Rhabdomyoma: A Surrogate Diagnosis of Tuberous Sclerosis Complex in a Newborn Baby: Case Report from Tikur Anbessa University Hospital

**DOI:** 10.4314/ejhs.v30i4.19

**Published:** 2020-07-01

**Authors:** Moges Tamirat, Beyene Ayalew

**Affiliations:** 1 Addis Ababa university, College of Health sciences, Pediatric cardiology unit. Cellphone-251-911405260, email-mogest98@yahoo.com , Addis Ababa Ethiopia; 2 Addis Ababa university , College of Health sciences, Pediatric neurology unit. Cellphone- 0911406533, email: Ayalewmg@yahoo.com, Addis Ababa Ethiopia.

**Keywords:** Cardiac rhabdomyoma, tuberous sclerosis complex, Hypomelanotic macules, sub-ependymal nodules and cortical tubers

## Abstract

**Background:**

Neonatal tuberous sclerosis complex is an autosomal dominant inherited disease characterized by high rate of neurological, cardiac and skin manifestations

**Case Presentation:**

We reported a 4 days old female neonate with respiratory distress, tachypnea, tachycardia and hypomelanotic macular lesions. Her chest X-ray and echocardiographic studies revealed cardiomegaly and multiple echogenic masses in the left and right ventricles, suggestive of cardiac rhabdomyoma. Furthermore, non-contrast brain magnetic resonance imaging revealed sub-ependymal nodules and cortical tubers. Therefore, a clinical diagnosis of neonatal tuberous sclerosis complex with heart failure was made. Then, the patient was initiated on diuretic treatment with oxygen by nasal catheter with subsequent improvement. Seizure was not occurred yet in the last three and half years of follow-up. Currently, the patient is thriving well with no symptoms

**Conclusion:**

Detection of prenatal or early neonatal age, cardiac rhabdomyoma is a useful clue to the diagnosis of tuberous sclerosis complex in neonates. Proper clinical evaluation of patients at the time of first contact prevents missing of findings such as skin macules and chest X-ray findings, which helped us to diagnose tuberous sclerosis complex in the present case

## Introduction

Tuberous sclerosis complex (TSC), an autosomal dominant inherited disease with an incidence of 1:6000 live births, causes benign tumors in brain, kidneys, heart and skin. Due to mutations in genes, TSC1 and TSC2, a benign tumor, rhabdomyoma grows in the heart. While 50-70 % of cases of TSC develop cardiac rhabdomyoma, over 80% of patients develop cortical tubers associated with epilepsy, autism or cognitive abnormality. Similarly, hypomelanotic macules, is observed in over 90% of patients at birth or in early infancy (1).

Infants with cardiac rhabdomyoma may present with arrhythmia, cyanosis, heart murmur, and/or heart failure. Echocardiography and cardiac MRI confirm the diagnosis (2). Although cardiac rhabdomyomas regresses spontaneously, some patients need medical treatment with a drug called Everolimus while others require surgery for arrhythmia control. Therefore, being cognizant of incidence of cardiac rhabdomyoma in TSC is vital for diagnosing TSC at an early age. Cardiac rhabdomyoma surrogating the diagnosis of TSC were reported in few instances globally and rarely in sub-Saharan Africa. Thus, the current report further discloses association between cardiac rhabdomyoma and TSC.

## Case Report

A 4 days old baby girl, presented with respiratory difficulty to a missionary hospital, was diagnosed clinically as early onset sepsis case. Despite antibiotic therapy, she remained in cardiorespiratory distress. Hence, she was referred to a tertiary hospital with chest X-ray evidence of cardiomegaly. The neonate was born from nonconsangeous marriage.

The patient weight, Height and Head circumference was 2,920gm, 47cm, and 34cm standardized to (25th–50th) (5th–10th) and (25th–50th) centile, respectively. RR-70/minute and AHR132bpm, BP-88/51mmHg, T-36.7oC and O2 sat 92% and 74% with and without oxygen respectively. Neonate had pink conjunctiva and the lung fields were clear. There was grade 3/6 holo-systolic murmur at tricuspid area, and liver age was tipped. There were four hypomelanotic macules over the buttock, legs and the trunk, the largest measuring 5 X 10mm in size.

**Figure 1:**
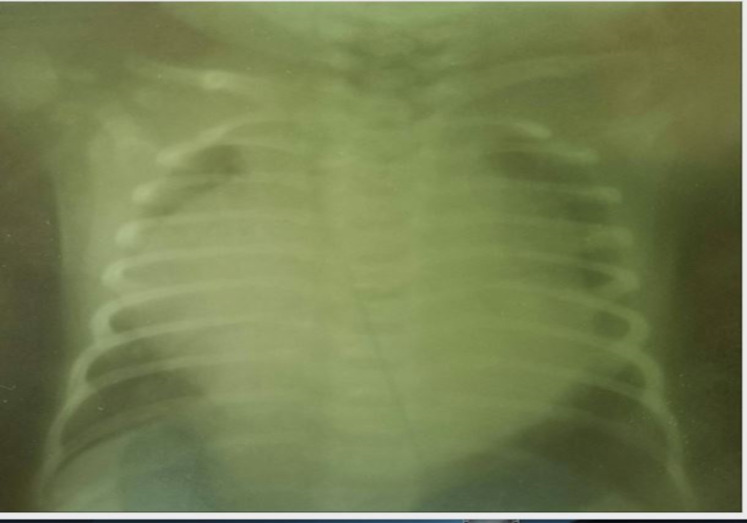
Chest X-ray film showing global cardiomegaly, with big heart compressing the lung fields bilaterally

**Figure 2:**
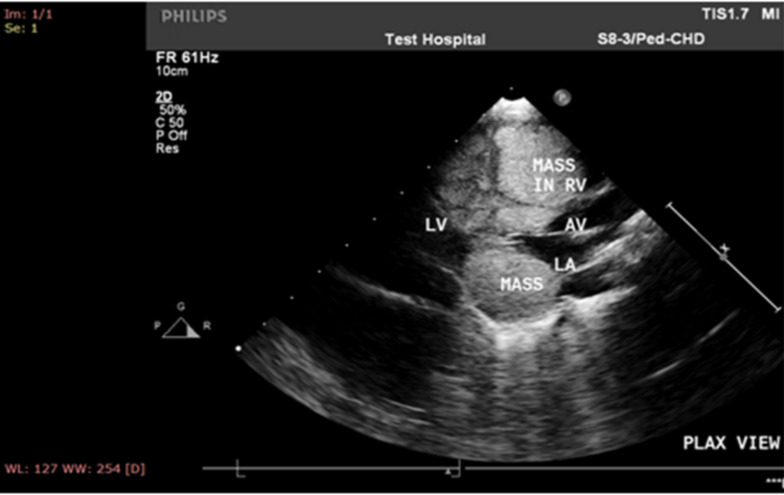
2D-echocardiographic image showing large echogenic mass with in the left atrium, the IVS and right ventricle

**Figure 3:**
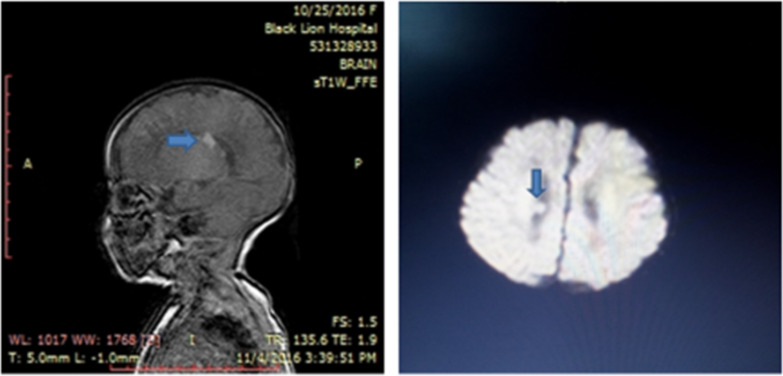
Brain magnetic resonance imaging (MRI) showing subependymal nodules and brain surface tubers

Chest X-ray showed an enlarged heart shadow compressing the lung fields bilaterally (Figure 1). 2D-echocardiography showed multiple, large echogenic masses in the right and left ventricular cavity, embedding the septum, left ventricular posterior wall and left atrium. The mass compressed the atrio-ventricular valves causing mitral and tricuspid regurgitation (Figure 2). Similarly, brain magnetic resonance imaging showed brain surface, sub-ependymal nodules and cortical tubers (Figure 3). Nevertheless, the EKG read as normal for age, trans-fontanel, and renal ultrasound studies were reported normal. Finally, the patient improved, the heart rate decreased from 160 to 104 bpm, RR decreased from 90 to 60/minute, and O2 sat increased from 74% to 98% at 3 litter oxygen. It was reported that the repeat echocardiography before discharge showed decrease in tumor size. The patient was discharged in a stable condition. The parents were counseled on future risks of epilepsy and accompanying cognitive abnormalities. Currently, the patient is 3 years and 6 months old, well thriving having follow-up at a missionary hospital in her locality.

## Discussion

This case is reported with the aim of broadening knowledge of the local staff in the specific settings, and the report strengthens the existing experience. The report showed the role of echocardiography in early diagnosis of TSC. We also emphasized the importance of the (ITSCG) in this report.

Although cardiac rhabdomyoma is earliest manifestation of neonatal TSC, isolated rhabdomyoma itself is a rare condition. The ITSCG in 2012, updated the diagnostic clinical criteria and includes 11 major and six minor features. As a result, “definite “TSC will be diagnosed when at least two major or one major plus two minor features are present. Conversely, “possible” diagnosis will be made with one major feature or ≥ 2 minor features (3). Our case had cardiac rhabdomyoma, hypomelanotic macules and subcortical brain tubers, fulfilling the ITSCG criteria. Peter E, et al reported hypomelanotic macules in 94%, sub-ependymal nodules in 90%, cardiac rhabdomyomas in 82% and tubers in 94% of their cases. In their report, seizure was noticed in only 15% of the cases before or at initial presentation. However, 73% of their cases developed epilepsy within the first year of life.

(4). Contrary to the above observations, our patient remained seizure free until her current age was 3 years and 6 months.

Our patient presented with symptoms of cardiorespiratory difficulty, which resolved without surgery. While most rhabdomyomas appear to regress spontaneously, some infants benefit from surgery if already caused obstructive symptoms.

Sciacca P et al reported that spontaneous reduction of rhabdomyomas was observed in their 32 of the 33 reported cases. For all patients, drug treatment was not believed necessary and the arrhythmia observed in 8 of the patients healed spontaneously (2). In the same manner, the improvement in our patient is attributed for decrease in tumor size. In one study, out of 11 fetal cardiac tumor cases, 5 survived and in all the surviving fetuses, the size of the mass regressed significantly (5). Although the aim of this report is to emphasize cardiac rhabdomyoma as surrogate diagnosis of TSC, having no follow-up images showing the regression of the tumor mass in the present report made the report incomplete. Future report should include follow-up images.

In conclusion, detection of prenatal or early neonatal age cardiac tumor is indisputably useful clue to the diagnosis of TSC in newborns. Proper clinical evaluation of patients at the time of first contact prevent missing of findings such as hypomelanotic macules and chest X-ray findings that helped us to diagnose the case.
